# Clinical features and recurrence predictors of histiocytic necrotizing lymphadenitis in Chinese children

**DOI:** 10.1186/s12969-024-00996-y

**Published:** 2024-06-11

**Authors:** Xiaoning Zhang, Xiuhong Jin, Xiangfeng Zhang, Yuelin Shen

**Affiliations:** 1 Respiratory Department, Children’s Hospital Affiliated to Zhengzhou University, Henan Children’s hospital, Zhengzhou Children’s Hospital, Zhengzhou, 450008 China; 2 Respiratory Department II, National Clinical Research Center for Respiratory Diseases, Beijing Children’s Hospital, Capital Medical University, National Center for Children’s Health, NO.56, Nanlishi Road, Beijing, 100045 China

**Keywords:** Histiocytic necrotizing lymphadenitis, Kikuchi-Fujimoto disease, Clinical features, Recurrence, Chinese children

## Abstract

**Objectives:**

To characterize the clinical features and to identify the predictors of recurrence of histiocytic necrotizing lymphadenitis (HNL) in Chinese children.

**Study design:**

This study retrospectively analyzed the clinical characteristics, laboratory and pathological findings, and recurrence status of children diagnosed with HNL at a single center in China from January 2018 to May 2023. Logistic regression analysis was employed to identify predictors of HNL recurrence.

**Results:**

181 Chinese children with histopathologically confirmed HNL were enrolled (121 males and 60 females). The mean age was 9.3 ± 2.9 years. The most prominent clinical features were fever (98.9%) and cervical lymphadenopathy (98.3%). Aseptic meningitis was the most frequent complication (38.5%), while hemophagocytic lymphohistiocytosis and autoimmune disease were rare (1.7% and 1.2%, respectively). Recurrence occurred in 12.7% of patients. Erythrocyte sedimentation rate (> 30 mm/h) was the significant predictors of HNL recurrence, with odds ratios of 6.107, respectively.

**Conclusion:**

Our study demonstrates that fever and cervical lymphadenopathy are the most frequent clinical manifestations of HNL in Chinese children, which often coexist with aseptic meningitis. HNL patients with risk factors require follow-up for recurrence.

Histiocytic necrotizing lymphadenitis (HNL), also known as Kikuchi-Fujimoto disease, is a rare and benign inflammatory condition of unknown etiology that mainly affects young adults and children of Asian origin. The incidence of HNL is not well established, but it is estimated to range from 0.3 to 4.6 cases per 100,000 population per year in different regions of the world, with higher rates in Asia and lower rates in Europe and America [1]. The most common clinical manifestation of HNL is localized lymphadenopathy, often accompanied by fever and other systemic symptoms [2]. The diagnosis of HNL is based on histopathological examination of lymph node biopsy. HNL usually has a self-limiting course and resolves spontaneously within a few weeks to months. However, some children may experience recurrence after achieving clinical remission [3]. The risk factors for HNL recurrence in pediatric patients have been poorly studied, and the recurrence rates vary across different countries and regions. We retrospectively reviewed 181 pediatric patients with histopathologically confirmed HNL at Children’s Hospital Affiliated to Zhengzhou University from January 2018 to May 2023. To our knowledge, this is the largest study to date on the clinical features and recurrence predictors of HNL in children.

## Methods

### Subjects

We retrospectively reviewed records of children treated between January 2018 and May 2023 at Children’s Hospital Affiliated to Zhengzhou University who met the following diagnostic criteria for HNL [4]: (1) Systemic symptoms or physical findings suggestive of HNL; (2) Histopathological confirmation of HNL by lymph node biopsy; (3) Exclusion of any other causes of lymphadenopathy. Recurrence was defined as additional episodes of febrile lymphadenopathy before or after the pathological diagnosis, excluding those due to infection, malignancy, or other definite etiologies [3]. The study was performed in line with the principles of the Declaration of Helsinki and was approved by the Ethics Committee of Children’s Hospital Affiliated to Zhengzhou University (2023-K-070).

### Clinical evaluation

We collected clinical data from all enrolled patients. This included demographic information, as well as clinical manifestations such as main symptoms (fever, headache, rash, joint pain, and oral ulcer), physical findings (lymph node enlargement, hepatomegaly, and splenomegaly), complications, treatments, and outcomes. Laboratory examinations included complete blood count (CBC), C-reactive protein (CRP), erythrocyte sedimentation rate (ESR), liver function tests, lactate dehydrogenase (LDH), serum ferritin (SF), anti-streptolysin O (ASO), lymphocyte subsets, and autoantibodies. We performed lumbar puncture and bone marrow aspiration to assist in diagnosing aseptic meningitis and hemophagocytic lymphohistiocytosis (HLH), respectively, and to rule out other causes of fever and lymphadenopathy. We recorded the size and number of the biopsied lymph nodes, the pathological results, and the recurrence status by reviewing the electronic medical records and by telephone follow-up.

### Statistical analysis

Statistical analyses were performed using SPSS software (version 22.0; SPSS, Chicago, IL, USA). The data were expressed as mean ± standard deviation (SD) or median (interquartile range, IQR) according to the distribution unless otherwise specified. The independent sample t-test was used for continuous variables, and the χ2 test (Yates correction) was used for categorical characteristics. If the data could not be transformed to approach normal distribution, a Mann-Whitney U test was applied. Binary logistic regression analysis was employed to evaluate recurrence predictors of HNL. A P value of less than 0.05 (two-tailed) was considered statistically significant.

## Results

### Cohort selection

A total of 209 children who met the inclusion criteria were recruited, of which 28 subjects were excluded due to Kawasaki disease (*n* = 5), infectious lymphadenitis (*n* = 3), lymphoma history (*n* = 1), or incomplete histopathological data (*n* = 19). Ultimately, a total of 181 subjects were included in the analysis. The mean age was 9.3 ± 2.9 years, with a male-to-female ratio of 2:1. The recurrence rate in our study was 12.7%. We divided all the patients into two groups based on their recurrence status. The comparison of clinical characteristics, laboratory examinations, complications and outcomes between the two groups of children is shown in Tables 1 and 2.

**Table 1:** Clinical characteristics of Chinese children with HNL at the onset of the first episode, stratified by recurrence status

Variables	Total (n = 181)	Non-recurrent HNL (n = 158)	Recurrent HNL (n = 23)	P value	
**Demographic data**					
Age, years	9.3 ± 2.9	9.4 ± 2.9	8.5 ± 2.4	0.192	
Sex, male/female	121/60	106/52	15/8	0.859	
Duration of follow-up, month	25.8 ± 12.1	25.3 ± 11.9	30.0 ± 13.1	0.113	
**Clinical manifestations**					
Fever, n (%)	179 (98.9%)	156 (98.7%)	23 (100%)	0.587	
Tmax ≥ 39℃, n (%)	175 (96.7%)	152 (96.2%)	23 (100%)	0.342	
Duration, day	25.6 ± 13.2	25.2 ± 11.9	28.0 ± 20.3	0.520	
Lymphadenopathy, n (%)	181 (100%)	158 (100%)	23 (100%)	NA	
Size, mm	25.9 ± 6.7	26.1 ± 6.6	25.0 ± 7.9	0.469	
Tenderness, n (%)	113 (62.4%)	100 (63.3%)	13 (56.5%)	0.531	
Location, n (%)					
Neck	178 (98.3%)	155 (98.1%)	23 (100%)	0.505	
Axilla	47 (26.0%)	42 (26.6%)	5 (21.7%)	0.621	
Abdomen	56 (30.9%)	48 (30.4%)	8 (34.8%)	0.670	
Groin	37 (20.4%)	30 (19.0%)	7 (30.4%)	0.203	
Headache	38 (21.0%)	32 (20.3%)	6 (26.1%)	0.521	
Rash	30 (16.6%)	27 (17.1%)	3 (13.0%)	0.626	
Joint pain	10 (5.5%)	7 (4.4%)	3 (13.0%)	0.091	
Oral ulcer	12 (6.6%)	10 (6.3%)	2 (8.7%)	0.670	
Hepatomegaly	17 (9.4%)	13 (8.2%)	4 (17.4%)	0.159	
Splenomegaly	18 (9.9%)	14 (8.9%)	4 (17.4%)	0.206	
**Complications, n (%)**					
Pneumonia	9 (5.0%)	7 (4.4%)	2 (8.7%)	0.379	
Aseptic meningitis (*n* = 96)	37 (38.5%)	32 (38.1%)	5 (41.7%)	0.812	
Transaminitis	91 (50.3%)	81 (51.3%)	10 (43.5%)	0.485	
HLH	3 (1.7%)	0 (0%)	3 (13.0%)	**< 0.001**	
JIA	1 (0.6%)	0 (0%)	1 (4.3%)	**0.009**	
SLE	1 (0.6%)	0 (0%)	1 (4.3%)	**0.009**	
**Treatments, n (%)**					
Glucocorticoid, n (%)	132 (72.9%)	114 (72.2%)	18 (78.3%)	0.538	
Glucocorticoid duration, day	60.0 (30.0, 90.0)	60.0 (30.0, 90.0)	54.0 (36.3, 108.8)	0.683	
**Outcomes**					
Defervescence after biopsy, n (%)	43 (23.8%)	38 (24.1%)	5 (21.7%)	0.808	
Defervescence time after biopsy, day	1.3 ± 0.5	1.3 ± 0.6	1.2 ± 0.4	0.597	
Defervescence after glucocorticoid, n (%)	129 (71.3%)	113 (71.5%)	16 (69.6%)	0.847	
Defervescence time after glucocorticoid, day	1.3 ± 0.6	1.3 ± 0.6	1.2 ± 0.5	0.645	

*Abbreviations* HLH = Hemophagocytic lymphohistiocytosis; JIA = Juvenile idiopathic arthritis; SLE = Systemic lupus erythematosus

**Table 2:** Laboratory and pathological examination of Chinese children with HNL at the onset of the first episode, stratified by recurrence status

Variables	Total (n = 181)	Non-recurrent HNL (n = 158)	Recurrent HNL (n = 23)	P value
**Complete blood count**, ***n*** (%)				
Leukopenia (WBC < 4 × 10^9^/L)	120 (66.3%)	111 (70.3%)	9 (39.1%)	**0.003**
Anemia (Hb < 110 g/L)	50 (27.6%)	42 (26.6%)	8 (34.8%)	0.411
Thrombocytopenia (PLT < 100 × 10^9^/L)	5 (2.8%)	5 (3.2%)	0	0.387
**Inflammatory markers, n (%)**				
CRP > 10 mg/L	53 (29.3%)	46 (29.1%)	7 (30.4%)	0.897
ESR > 30 mm/h	106 (58.6%)	86 (54.4%)	20 (87.0%)	**0.003**
LDH > 300 U/L, *n* = 170	149 (87.6%)	130 (87.8%)	19 (86.4%)	0.845
SF > 500ng/ml, *n* = 124	30 (24.2%)	25 (23.6%)	5 (27.8%)	0.701
**Positive autoantibodies, n (%)**				
ANA, *n* = 162	15 (9.3%)	14 (9.9%)	1 (4.8%)	0.446
RF-IgM, *n* = 110	24 (21.8%)	21 (22.1%)	3 (20.0%)	0.854
RF-IgA, *n* = 110	11 (10.0%)	10 (10.5%)	1 (6.7%)	0.643
Anti-RA33-IgG, *n* = 113	15 (13.3%)	12 (12.2%)	3 (20.0%)	0.410
**Pathological findings, n (%)**				
Necrosis	181 (100%)	158 (100%)	23 (100%)	NA
Histiocytosis	181 (100%)	158 (100%)	23 (100%)	NA
Positive CD68	181 (100%)	158 (100%)	23 (100%)	NA
Positive CD123	153 (91.7%)	133 (91.1%)	20 (95.2%)	0.731

*Abbreviations* ALT = Alanine aminotransferase; ANA = Anti-nuclear antibody; Anti-RA33-IgG = Anti-rheumatoid arthritis 33 antibody-IgG; CRP = C-reactive protein; ESR = Erythrocyte sedimentation rate; Hb = Hemoglobin; LDH = Lactate dehydrogenase; PLT = Platelet; RF = Rheumatoid factor; SF = Serum ferritin; WBC = White blood cell count

### Clinical manifestations

The most common symptoms in children with HNL were fever and lymphadenopathy. Fever occurred in 98.9% of patients, reaching a maximum temperature above 38.5℃ and lasting for 25.6 ± 13.2 days. Lymphadenopathy affected all patients, with the cervical region being the most frequently involved (98.3%), followed by the abdominal (30.9%), axillary region (26.0%) and inguinal (20.4%). Lymph node pain was reported by 62.4% of patients. Other uncommon manifestations included headache (21.0%), rash (16.6%), splenomegaly (9.9%), hepatomegaly (9.4%), oral ulcer (6.6%), and joint pain (5.5%). There was no significant difference between the groups regarding fever duration, headache, rash, oral ulcer, headache and joint pain.

Of the 181 patients, three (1.7%) met the diagnostic criteria for hemophagocytic lymphohistiocytosis (HLH); one (0.6%) developed recurrent bilateral knee pain six months post-NHL diagnosis and was diagnosed with juvenile idiopathic arthritis (JIA); and one was diagnosed with systemic lupus erythematosus (SLE) 17 months after symptom onset.

### Laboratory examinations

All patients underwent CBC and blood biochemistry tests. Leukopenia was found in 66.3% of patients. Autoantibody profile was performed on most of the patients, and the positive rates of anti-nuclear antibody (ANA), rheumatoid factor (RF)-IgM, RF-IgA, and anti-rheumatoid arthritis 33 antibody-IgG (anti-RA33-IgG) were 9.3%, 21.8%, 10.0%, and 13.3%, respectively.

WBC (< 4 × 10^9^/L), ESR (> 30 mm/h) showed significant differences between the recurrent group and the non-recurrent group (*p* = 0.003 for both). No significant differences were observed between the two groups in other laboratory parameters. Of the 96 patients who underwent lumbar puncture, 37 (38.5%) were diagnosed with aseptic meningitis. Bone marrow puncture was performed on 132 patients, and only one patient (0.8%) showed decreased proliferation. This patient unfortunately succumbed to HLH. The remaining patients (99.2%) showed active proliferation.

### Treatment and outcome

Of the 181 children with HNL, three (1.7%) had normal body temperature before biopsy and 43 (23.8%) became afebrile after biopsy. We recommended glucocorticoid therapy for all 135 patients who remained febrile after biopsy. However, three (1.7%) declined treatment due to concerns about drug side effects, while 132 (72.9%) received glucocorticoid therapy. Of these, 129 patients responded well to the treatment. There was no significant difference in glucocorticoid use or duration between the two groups of HNL children. The follow-up period ranged from 6 months to 5 years. During this time, 23 children (12.7%) experienced at least one episode of HNL recurrence. Among them, 16 cases had one recurrence, 5 cases had two recurrences, and 2 cases had three recurrences. The average time from disease onset to the first recurrence was 11.3 ± 8.1 months (range: 2.5–28 months). The interval between the second recurrence and the first recurrence was 11.7 ± 8.2 months (range: 3–25 months). The intervals between the third recurrence and the second recurrence were 8 months and 1 months, respectively, for only two patients. Of these cases, 13 underwent lymph node biopsy again with histopathological confirmation, while 10 had clinical features of fever, lymphadenopathy, leukopenia, elevated ESR, and improved after glucocorticoid treatment (clinically suspected HNL recurrence). The remaining 87.3% of children did not experience recurrence. Three children with HLH were treated with glucocorticoids and immunoglobulin; two improved while the other died of disseminated intravascular coagulation and multiple organ failure.

### Risk factors for recurrent HNL

We conducted a binary logistic regression analysis with the presence of HLH, JIA, SLE, as well as WBC (< 4 × 10^9^/L) and ESR (> 30 mm/h) as independent variables that were significant in univariate analysis. The results showed ESR (> 30 mm/h) were the significant predictors of HNL recurrence, with odds ratios of 6.107 (Table 3).

**Table 3:** Logistic regression analysis of risk factors associated with HNL recurrence

	B	S.E	Waldχ^2^	P value	OR	95% CI
ESR > 30 mm/h	1.809	0.771	5.503	**0.019**	6.107	1.347–27.692

*Abbreviations* ESR = Erythrocyte sedimentation rate

## Discussion

To the best of our knowledge, this is the largest study to date on the clinical features and recurrence predictors of HNL in children. HNL is mainly prevalent in Asia, especially in East Asian regions such as Japan, Korea, and China. However, isolated cases have been reported in other continents and regions, such as America, Africa, Europe, New Zealand, and Australia. HNL is more common in young adult and elderly pediatric patients [5]. In this study, the mean age of the children with HNL was 9.3 ± 2.9 years, and 91.2% of them were older than 6 years, which is consistent with previous reports. However, clinical manifestations may differ between children and adults. This disease is more prevalent among adult females but more common among male children [6, 7]. The male-to-female ratio in this study was 2:1, which is consistent with previous reports. Fever and unilateral tenderness of cervical lymph nodes are the most common symptoms of HNL. The reported fever rate for adult patients with HNL ranges from 25 to 87%, and the duration of fever may vary from a few days to several weeks depending on the severity of the disease and response to treatment. In contrast, children with HNL often have a higher proportion and longer duration of fever [8]. In our study, as many as 98.9% of patients had fever, with a mean duration of 25.6 ± 13.2 days. Therefore, the duration of fever may be a useful parameter for distinguishing between pediatric and adult HNL. All children in our study had Lymphadenopathy, with cervical lymph node enlargement being the most common (98.3%). The incidence of cervical lymph node involvement in our study is consistent with that reported in previous studies on pediatric HNL (95%) [4].

Several previous studies [3, 9] have shown a close association between HNL and autoimmune diseases and have even reported an “overlap” phenomenon between them. HNL may precede, coincide with or follow the onset of autoimmune diseases. According to Jung et al. [3] positive antinuclear antibody (ANA) could be a risk factor for the progression of HNL to autoimmune diseases. However, in our study, only one child who tested positive for ANA developed systemic lupus erythematosus 17 months after symptom onset. Shahrokh et al. [10] reported a case of HNL associated with rheumatoid arthritis. In our study, we observed a similar case of a child who had lateral knee pain 10 months before being diagnosed with HNL and recurrent bilateral knee pain 6 months after the diagnosis. The child was later diagnosed with systemic JIA and treated with methotrexate and celecoxib, which resulted in improvement of joint pain.

Aseptic meningitis is a rare complication of HNL that affects 2.2–9.8% of patients [11]. The exact mechanism of HNL-associated aseptic meningitis is not clear, but it may be related to the immune-mediated inflammatory response triggered by HNL. The diagnosis of aseptic meningitis in HNL patients is based on clinical manifestations and CSF analysis. CSF usually shows increased lymphocytes, normal or slightly elevated protein levels, and normal glucose levels. In this study, the incidence of aseptic meningitis was 38.5% (37/96), which is significantly higher than that reported in previous studies. This may be related to the fact that we performed lumbar puncture examination in all HNL children regardless of whether they had neurological symptoms or not. The treatment of aseptic meningitis in HNL patients is mainly supportive. The prognosis is generally good, and neurological symptoms can resolve spontaneously within several weeks to months, with a low recurrence rate. In 41 cases of HNL combined with aseptic meningitis summarized by Song et al. [12], 41.5% of patients recovered without steroid treatment, while 58% of patients received steroid treatment and 79% of them fully recovered.

HNL may be complicated by HLH, a condition that can have various etiologies, such as genetic, infectious, neoplastic, rheumatic or other factors. The prevalence of HNL-HLH differs across studies. Yang et al. [13] reported that HNL-HLH was very rare in children. However, Shen et al. [14] found that HLH was more frequent in children with HNL than previously thought, especially in those with rash. In our study, only 3 children (1.7%) had HNL-HLH, much lower than the report by Shen et al. [12], which may reflect the different severity of the disease in the two studies. HNL-HLH can be managed with glucocorticoids and immunoglobulin. For patients who are resistant to steroids, short-term chemotherapy such as VP16 can be administered, but the prognosis is uncertain. Zhou et al. [15] reported a mortality rate of 7.0% at initial treatment. In our study, despite intensive treatment, the prognosis of three patients differed significantly. Two children responded well to glucocorticoids and immunoglobulin, while the other child died of disseminated intravascular coagulation and multiple organ failure.

Previous studies have shown that HNL can recur, with recurrence rates ranging from 10 to 42.4% in children [3, 6, 16]. In our study, 12.7% of patients had recurrence, which is consistent with the findings of Selvanathan et al. (12.2%) [16] and Jung et al. (13.2%) [3]. Zhang et al. [17] reported a higher recurrence rate of 20.3% in 118 children with HNL, which may be due to their longer follow-up duration (median 48 months, range 12–84 months) compared to our study (median 25.8 months, range 6–60 months). However, Yoo et al. [6] also reported a higher recurrence rate of 42.4% in 33 children with HNL, but their follow-up duration (median 21 months, range 1–48 months) was shorter than that of our study. This may be due to the different criteria of HNL recurrence between the two studies. Yoo et al. [6] considered HNL recurrence as the reappearance of clinical symptoms without histopathological confirmation, while in our study, 56.5% of recurrent patients (13/23) underwent repeated lymph node biopsy. Currently, the risk factors for HNL recurrence in children are poorly understood, and the etiology of HNL recurrence remains elusive. In our study, we found that the recurrence group had a higher ESR than the non-recurrence group, which is in agreement with the findings of Song et al. [18], indicating that an excessive inflammatory response may be a key factor in the pathogenesis of HNL recurrence.

The optimal management of HNL remains controversial. In our study, 23.8% of patients became afebrile after lymph node biopsy, which might be attributed to the removal of lesions that halted the disease progression to some extent. Several studies have demonstrated the significant efficacy of glucocorticoid therapy for HNL [14, 19]. However, considering the adverse effects of glucocorticoid, Kang et al. recommended short-term administration for children with severe or prolonged illness [4]. In our study, the body temperature of HNL patients normalized within 1–3 days after receiving glucocorticoid therapy, suggesting that it can ameliorate the symptoms of patients. However, there was no statistically significant difference in the rate and duration of glucocorticoid use between the recurrence and non-recurrence groups, indicating that long-term therapy cannot prevent recurrence. Lin et al. claimed that hydroxychloroquine therapy for HNL was as effective as glucocorticoid therapy [20]. In our study, only one patient received combined therapy with both drugs, and the outcome was favorable. There are also reports that biologics such as recombinant human IL-1 receptor antagonists can alleviate symptoms in patients with recurrent HNL [21].

This study had some limitations. First, the follow-up duration was relatively short, precluding the evaluation of long-term prognosis of children with HNL. Moreover, since HNL may predispose to SLE, JIA, or other autoimmune diseases, long-term follow-up is also needed. Second, retrospective studies may have selection bias. Third, the patients were mostly from Central China, and this was a single-center study. We hope that future studies will be multi-center, larger-scale, and prospective to further confirm our findings.

## Conclusions

In summary, our study demonstrates that fever and cervical lymphadenopathy are the most frequent clinical manifestations of HNL in Chinese children, which often coexist with aseptic meningitis. The overall prognosis of HNL is favorable. However, HNL patients with risk factors require follow-up for recurrence. ESR was the independent predictors of HNL recurrence, suggesting that an excessive inflammatory response may play a significant role in the pathogenesis of HNL recurrence. Further prospective studies with large samples from multiple centers and long-term follow-up are needed to understand the clinical characteristics and prognosis of children with HNL.

## Data Availability

Data sharing not applicable to this article as no datasets were generated or analyzed during the current study.

## Abbreviations

ALT: Alanine aminotransferase
ANA: Anti-nuclear antibody
ANC: Absolute neutrophil count
Anti-RA33-IgG: Anti-rheumatoid arthritis 33 antibody-IgG
ASO: Anti-streptolysin O
AST: Aspartate aminotransferase
CBC: Complete blood count
CRP: C-reactive protein
ESR: Erythrocyte sedimentation rate
HLH: Hemophagocytic lymphohistiocytosis
HNL: Histiocytic necrotizing lymphadenitis
JIA: Juvenile idiopathic arthritis
LDH: Lactate dehydrogenase
LC: Absolute lymphocyte count
PLT: Platelet
RF: Rheumatoid factor
SF: Serum ferritin
SLE: Systemic lupus erythematosus
WBC: White blood cell count
